# Long-Term Grazing Exclusion Improves the Composition and Stability of Soil Organic Matter in Inner Mongolian Grasslands

**DOI:** 10.1371/journal.pone.0128837

**Published:** 2015-06-09

**Authors:** Chunyan Wang, Nianpeng He, Jinjing Zhang, Yuliang Lv, Li Wang

**Affiliations:** 1 Key Laboratory of Ecosystem Network Observation and Modeling, Institute of Geographic Sciences and Natural Resources Research, Chinese Academy of Sciences, Beijing, 100101, China; 2 College of Geographical Science, Southwest University, Chongqing, 400715, China; 3 College of Resource and Environmental Science, Jilin Agricultural University, Changchun, 130118, China; Tennessee State University, UNITED STATES

## Abstract

Alteration of the composition of soil organic matter (SOM) in Inner Mongolian grassland soils associated with the duration of grazing exclusion (GE) has been considered an important index for evaluating the restoring effects of GE practice. By using five plots from a grassland succession series from free grazing to 31-year GE, we measured the content of soil organic carbon (SOC), humic acid carbon (HAC), fulvic acid carbon (FAC), humin carbon (HUC), and humic acid structure to evaluate the changes in SOM composition. The results showed that SOC, HUC, and the ratios of HAC/FAC and HAC/extractable humus carbon (C) increased significantly with prolonged GE duration, and their relationships can be well fitted by positive exponential equations, except for FAC. In contrast, the HAC content increased logarithmically with prolonged GE duration. Long-term GE enhanced the content of SOC and soil humification, which was obvious after more than 10 years of GE. Solid-state ^13^C nuclear magnetic resonance spectroscopy showed that the ratios of alkyl C/O-alkyl C first decreased, and then remained stable with prolonged GE. Alternately, the ratios of aromaticity and hydrophobicity first increased, and then were maintained at relatively stable levels. Thus, a decade of GE improved the composition and structure of SOM in semiarid grassland soil and made it more stable. These findings provide new evidence to support the positive effects of long-term GE on soil SOC sequestration in the Inner Mongolian grasslands, in view of the improvement of SOM structure and stability.

## Introduction

Soil organic matter (SOM) plays important roles in retaining and supplying plant nutrients, and in improving soil aggregation and erodibility [1,2]. SOM, as the largest carbon (C) pool in terrestrial ecosystems, has been commonly divided into active, slow, and passive C fractions according to the turnover time [3]. Six et al. [4] divided SOM into protected or unprotected fractions to explore the underlying mechanisms of decomposition. These fractions have some overlap in stabilization mechanisms, such that the unprotected pool represents the active fractions and part of the slow pool, and the biochemically protected pool is comparable to the passive pool to some extent.

Some studies have investigated changes in SOM composition and stability in agriculture ecosystems by mainly evaluating humic substances and other organic macromolecules [5]. SOM components related to soil quality are closely associated with soil humified fractions [6], which can improve soil buffering capacity, moisture retention, and micronutrient supply [7]. Changes in soil humus are supposed to be the most effective component and represent the stability of soil structure and resistance to erosion [8]. According to its classical classification, soil humus can be divided into humic acid (HA), fulvic acid (FA), and humin (HU). Different components of soil humus have specific contributions towards soil fertility according to their humus composition and chemical structure [9].

Few studies have investigated changes in SOM composition and structure, although soil C sequestration resulting from land-use change or management of forest and grassland has been evaluated [10,11]. In Inner Mongolian grasslands (78.8 × 10^6^ ha), the practice of grazing exclusion (GE) has been deemed as an effective approach to restore these degraded grasslands. At the same time, some studies have demonstrated that long-term GE has tremendous potential for increasing soil C and nitrogen storage in temperate grasslands in northern China [12–16]. However, it is still unclear how SOM composition and structure change dynamically with the duration of GE.

In this study, we used a grassland restoration chronosequence with five GE durations (0–31 year) in Inner Mongolia to investigate the dynamics of SOM composition after GE. Furthermore, we used solid-state ^13^C cross-polarization magic spinning nuclear magnetic resonance spectroscopy (CPMAS NMR) to explore changes in HA structure. The main objectives of the present study were to: 1) investigate the influences of long-term GE on SOM composition in semiarid grassland soils, and 2) explore changes in SOM stability with long-term GE.

## Material and Methods

### Study sites

The experimental plots belong to typical temperate grassland at the Inner Mongolia Grassland Ecosystem Research Station (IMGERS) of the Chinese Academy of Sciences (43°33′N,116°40′E), which has a typical semi-arid continental climate. The mean annual temperature is 1.1°C. The annual precipitation is approximately 345 mm, 70% rainfall occurring in June, July, and August. The soil is chestnut, which is equivalent to Calcic Orthic Aridisol in the US soil taxonomy classification system, and it developed from Aeolian sediments. The soils are characterized by rich sand content with the range of sand from 60% to 75% [17]. The vegetation consists predominantly of grassland plants, such as *Leymus chinensis* (44.5%, relative biomass), *Stipa grandis* (34.0%), and *Cleistogenes squarrosa* (8.7%) [15].

Five experimental plots were selected based on the preexisting experimental plots of IMGERS. The plots were designated as GE0, GE4, GE7, GE11, and GE31. Plot GE0 had been exposed to long-term grazing by sheep and was in a slightly degraded condition in terms of plant community and diversity. Plots GE4, GE7, GE11, and GE31 were established in 2008, 2004, 1999, and 1979, respectively, by fencing off a section of previous grazing grasslands. These GE plots ranged from 0.8 ha to 24 ha in area, and had similar vegetation and topography across a 2-km area. Changes in soil properties in these plots (as presented in Table 1) therefore mainly resulted from the influence of grazing intensity and GE duration on new organic matter input by plants and SOM turnover.

**Table 1:** Changes in the selected soil properties in the grazing-exclusion grassland chronosequence.

Grassland type	Aboveground biomass (gm^–2^)	Litter (g m^–2^)	SOC^‡^ (g kg^–1^)	TN (g kg^–1^)	TP (g kg^–1^)	PH
GE0^†^	60.28 ± 20.60 ^b^ ^§^	30.53 ± 13.83^c^	14.36 ± 1.26^c^	1.41 ± 0.01^b^	0.22 ± 0.02^c^	8.16 ± 0.29^a^
GE4	162.25 ± 14.97^a^	62.85 ± 7.51^b^	14.31 ± 0.61^c^	1.60 ± 0.01^a^	0.27 ± 0.01^b^	8.07 ± 0.11^a^
GE7	166.18 ± 13.27^a^	75.17 ± 12.37^b^	15.03 ± 0.96^c^	1.64 ± 0.02^a^	0.30 ± 0.01^a^	7.92 ± 0.16^a^
GE11	171.64 ± 9.64^a^	82.84 ± 18.27^b^	17.23 ± 1.27^b^	1.72 ± 0.01^a^	0.29 ± 0.01^a^	7.66 ± 0.19^a^
GE31	148.93 ± 41.27^a^	121.12 ± 32.69^a^	19.95 ± 0.27^a^	1.42 ± 0.07^c^	0.28 ± 0.01^b^	7.19 ± 0.29^b^
F	20.508	14.947	18.731	210.606	50.698	4.84
P	<0.001	<0.001	<0.001	<0.001	<0.001	0.007

^†^ GE0, free grazing; GE4, 4-year grazing exclusion; GE7, 7-year grazing exclusion; GE11, 11-year grazing exclusion; GE31, 31-year grazing exclusion.

^‡^ SOC, soil organic carbon; TN, Soil total nitrogen; TP, Soil total phosphorus.

^§^ Data were represented as mean ± SD (n = 4). The same superscript letters within each column indicated no significant difference at *P* < 0.05.

### Field sampling

In each experimental plot, an east-west transect was established with four equal-sized replicate blocks (20 × 20 m each). Field sampling was conducted in July 2011. In each block, one sampling quadrat (each 1 m × 1 m) was first established to investigate aboveground biomass with all the plant species combined. Litter was subsequently collected. In each block, approximately 10 soil cores were taken randomly to a depth of 20 cm using a soil auger (8 cm in diameter), and mixed as a sample. Each sample was air-dried in a ventilation room, sieved using 2-mm sieves, and cleared of visible roots and organic debris by hand for further analysis.

### Laboratory analysis

The content of organic C in all samples was measured by using the modified Mebius method [18]. Total soil nitrogen (TN) was measured with a modified Kjeldahl wet digestion procedure [19], using a 2300 Kjeltec Analyzer Unit (FOSS Tecator, Hoganas, Sweden). Total phosphorus (TP) was determined by the ammonium molybdate method after persulfate oxidation [20]. Soil pH was determined using a pH meter and a slurry of soil mixed with distilled water (1:2.5). In this study, the measurements for soil properties were conducted in four replicates.

### Humus composition analysis

Soil humus composition was analyzed as proposed by Kumada [21] with minor modifications [22]. Briefly, a 5-g soil sample was passed through a 60-mesh sieve and placed in 100 mL centrifuge tubes. Distilled water (80 mL) was then added to each tube and the tubes were shaken for 1 h at 70°C in a thermostatic water bath oscillator. The mixture was centrifuged at 3500 r min^–1^ for 15 min, and the supernatant was discarded. The residue, which was the precipitate in the centrifuge tube, was washed twice with distilled water. Subsequently, a 30 mL mixture of 0.1 mol L^–1^ NaOH and 0.1 mol L^–1^ sodium pyrophosphate was added to the soil residue (pH 13), shaken for 1 h at 70°C and then centrifuged at 3500 r min^–1^ for 15 min. The supernatant was filtered into a 50 ml volumetric flask. The residue was washed twice with 20 mL of the above mixture (10 mL every time). The supernatant from the second centrifugation step was also filtered into the same 50 mL volumetric flask to a final volume of 50 mL. The solution contained extractable humic substances. The residue in the centrifuge tube was incubated with distilled water at 55°C and passed through a 60-mesh sieve to provide HU. To 30 mL of the humic substance solution, 0.5 mol L^–1^ H_2_SO_4_ was added and the pH was adjusted to 1.0–1.5. The mixture was subjected to 60–70°C for 1.5 h, and then left overnight. The following day, the solution was filtered into a 50 mL volumetric flask to obtain FA after the volume was determined. The precipitate on the filter paper was washed three times with 0.25 mol L^–1^ H_2_SO_4_ and dissolved in a 50 mL volumetric flask using 0.05 mol L^–1^ NaOH to obtain HA, after adding distilled water to volume. The C contents of extractable humic substances, HU (HUC), and HA (HAC) were determined by the K_2_Cr_2_O_7_ method [18], whereas the C content of FA (FAC) was calculated by subtracting HAC from the extractable humus substance content [23].

### Humic acid measurement

Isolation and purification of HA were conducted following previous described methods [24,25] with minor modifications [26]. Briefly, 100 g of the soil sample was first suspended in distilled water and 0.05 mol L^–1^ HCl to remove poorly decomposed light fractions and carbonates. The soil samples were then extracted using a solution of 0.1 mol L^–1^ NaOH and 0.1 mol L^–1^ Na_4_P_2_O_7_ with 5% (w/v) Na_2_SO_4_·10H_2_O at 25°C for 48 h. The extraction procedure was repeated three times on the residues until the supernatant was colorless. The combined alkaline supernatants were acidified to pH 1.0 with 6 mol L^–1^ HCl to separate HA. After three cycles of dissolution in 0.1 mol L^–1^ NaOH and re-precipitation with 6 mol L^–1^ HCL, HA was shaken five times in a 0.5% (v/v) HCl-HF solution, dialyzed against distilled water until it was Cl-free, and finally freeze-dried.

The solid-state ^13^C CPMAS NMR spectra were used to measure SOM composition on a Bruker (Switzerland) spectrometer operating at 100.61 MHz, equipped with a 4 mm probe head. The conditions were as follows: spinning rate 5 kHz, contact time 4 ms, recycle delay time 0.5 s, line broadening 100 Hz, and zero-filling 3072 data points. The spinning side band was corrected according to Conte et al. [27]. According to the main chemical shift regions, spectra were divided into four regions [28]: alkyl C (0–50 ppm), O-alkyl C (50–110 ppm), aromatic C (110–160 ppm), and carbonyl C (160–200 ppm). As the methods described by Dai et al. [29] and Zhang et al. [26], aromaticity and hydrophobicity were calculated as follows:

$$\text{Aromaticity }\left(\text{\%}\right)=\left[\frac{\text{Aromatic C}}{\text{Aromatic C }+\text{ Alkyl C }+\text{ O}–\text{alkyl C}}\right]\times 100$$ (1)

$$\text{Hydrophobicity }=\;\frac{\text{Alkyl C }+\text{ Aromatic C}}{\text{O}–\text{alkly C }+\text{ Carbonyl C}}$$ (2)

### Statistical analyses

One-way analysis of variance (one-way ANOVA) with Duncan tests was used to evaluate the differences in soil properties and SOM composition among different grasslands. Pearson correlations were evaluated between different SOM compositions. Regression analyses were conducted to test the relationships between SOM composition and GE duration. Statistical significance was defined as *P* = 0.05. All statistical analyses were performed using SPSS (version 13.0).

## Results

### Changes in soil properties

**Fig 1:**
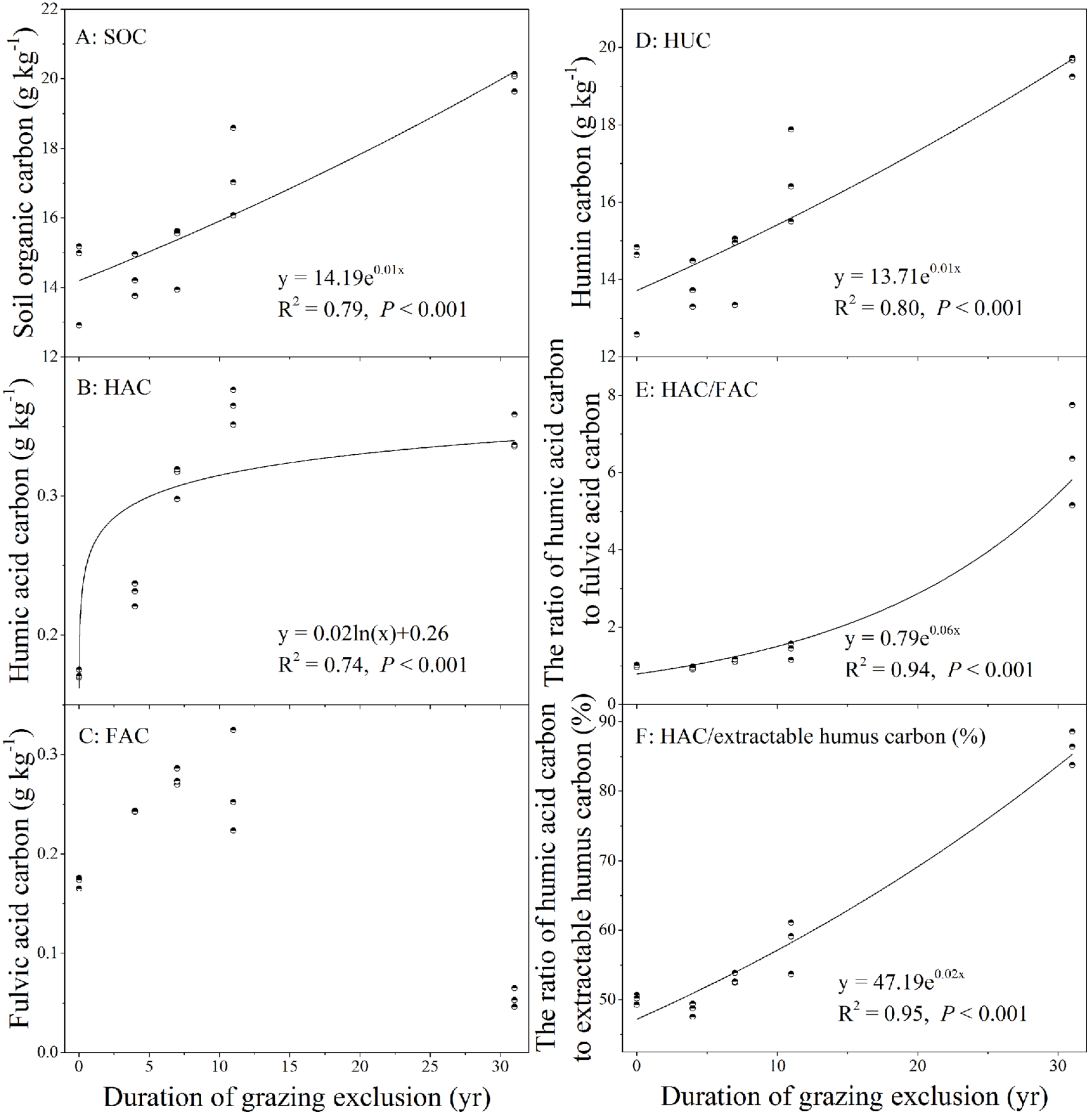
Relationships between soil organic carbon (SOC), humic acid carbon (HAC), fulvic acid carbon (FAC), humic acid carbon (HUC), HAC/FAC, HAC/extractable humus carbon with the duration of grazing exclusion.

There were significant increases in the aboveground biomass, litter, SOC, TN, and TP after GE, and these parameter were significantly different between grazing grassland (GE0) and long-term GE grasslands (all *Ps* < 0.001; Table 1). Moreover, the content of SOC increased exponentially with the duration of GE (R^2^ = 0.79, *P* < 0.001)(Fig 1A). The contents of TN and TP in soils first increased and then decreased to some extent with prolonged GE. Soil pH decreased from 8.10 in GE0 to 7.19 in GE31, but it was not significantly different among the 4 GE grasslands.

### Changes in SOM composition

The content of different SOM components varied significantly among the five plots (all *Ps* < 0.001; Table 2). In detail, the content of HAC increased from 0.17 g kg^–1^ in GE0 to 0.36 g kg^–1^, and the relationship between HAC and GE duration was be well fitted by a logarithmic equation (R^2^ = 0.74, *P* < 0.001) (Fig 1B). Additionally, HUC, HAC/extractable humus C, and HAC/FAC all increased exponentially with the duration of GE (R^2^ = 0.80, *P* < 0.001 for HUC; R^2^ = 0.95, *P* < 0.001 for HAC/extractable humus C; R^2^ = 0.94, *P* < 0.001 for HAC/FAC). In contrast, FAC did not have a similar pattern, as it first increased and then decreased to some extent with the prolonged GE (Table 2).

**Table 2:** Changes in the SOM composition along the grazing-exclusion grassland chronosequence.

Grassland type	HAC^‡^ (g kg^–1^)	FAC (g kg^–1^)	HUC (g kg^–1^)	Extractable humus C (g kg^–1^)	HAC/FAC	HAC/ extractable humus C (%)
GE0^†^	0.17 ± 0.01^e^ ^§^	0.17 ± 0.01^b^	14.02 ± 1.25^c^	0.34 ± 0.01^c^	1.00 ± 0.03^b^	50.04 ± 0.70^cd^
GE4	0.23 ± 0.01^d^	0.24 ± 0.01^a^	13.83 ± 0.60^c^	0.47 ± 0.01^b^	0.95 ± 0.04^b^	48.58 ± 0.97^d^
GE7	0.31 ± 0.01^c^	0.28 ± 0.01^a^	14.45 ± 0.96^c^	0.59 ± 0.02^a^	1.13 ± 0.03^b^	52.96 ± 0.77^c^
GE11	0.36 ± 0.01^a^	0.27 ± 0.05^a^	16.60 ± 1.20^b^	0.63 ± 0.06^a^	1.39 ± 0.21^b^	57.96 ± 3.84^b^
GE31	0.34 ± 0.01^b^	0.05 ± 0.01^c^	19.55 ± 0.26^a^	0.40 ± 0.01^c^	6.42 ± 1.30^a^	86.25 ± 2.41^a^
F	180.731	43.755	20.219	48.249	49.193	160.792
P	<0.001	<0.001	<0.001	<0.001	<0.001	<0.001

^†^ GE0, free grazing; GE4, 4-year grazing exclusion; GE7, 7-year grazing exclusion; GE11, 11-year grazing exclusion; GE31, 31-year grazing exclusion.

^‡^ HAC, Humic acid carbon; FAC, Fulvic acid carbon; HUC, Humin carbon; Extractable humus C, Extractable humus carbon;HAC/FAC, The ratio of humic acid carbon to fulvic acid carbon; HAC/extractable humus C, The ratio of humic acid carbon to extractable humus carbon.

^§^ Data were represented as mean ± SD (n = 3). The same superscript letters within each column indicated no significant difference at *P* < 0.05.

HUC was not significantly different among GE0, GE4, and GE7, but it increased exponentially with the duration of GE (R^2^ = 0.80, *P* < 0.001; Fig 1 and Table 2). The ratio of HAC/extractable humus C was lowest in GE4 (48.58%) and highest in GE31 (86.25%), and it increased exponentially with the duration of GE (R^2^ = 0.95, *P* < 0.001; Fig 1F). Furthermore, the ratios of HAC/FAC also increased exponentially with the duration of GE (R^2^ = 0.94, *P* < 0.001; Fig 1E).

### Relationships among C content in different components

SOC, HAC, and HUC were positively correlated with each other (Table 3). Moreover, HAC/extractable humus C and HAC/FAC had significantly positive correlations with SOC. HUC, HAC/extractable humus C, and HAC/FAC had significantly positive correlations with each other, whereas FAC showed negative correlations with other components (Table 3).

**Table 3:** Pearson correlation of organic carbon among different SOM components.

	SOC	HAC	FAC	HUC	HAC/FAC	HAC/extractable humus C
SOC^†^	1					
HAC	0.708**	1				
FAC	-0.557*	0.001	1			
HUC	0.999**	0.676**	-0.595*	1		
HAC/FAC	0.823**	0.464	-0.848**	0.840**	1	
HAC/ Extractable humus C	0.870**	0.575*	-0.813**	0.883**	0.973**	1

^†^ SOC, soil organic carbon; HAC, humic acid carbon; FAC, Fulvic acid carbon; HUC, Humin carbon; Extractable humus C, Extractable humus carbon; HAC/FAC, The ratio of humic acid carbon to fulvic acid carbon; HAC/extractable humus C, The ratio of humic acid carbon to extractable humus carbon.

* *P* < 0.05 and

**P < 0.01.

### Changes in the structure of humic acid

**Fig 2:**
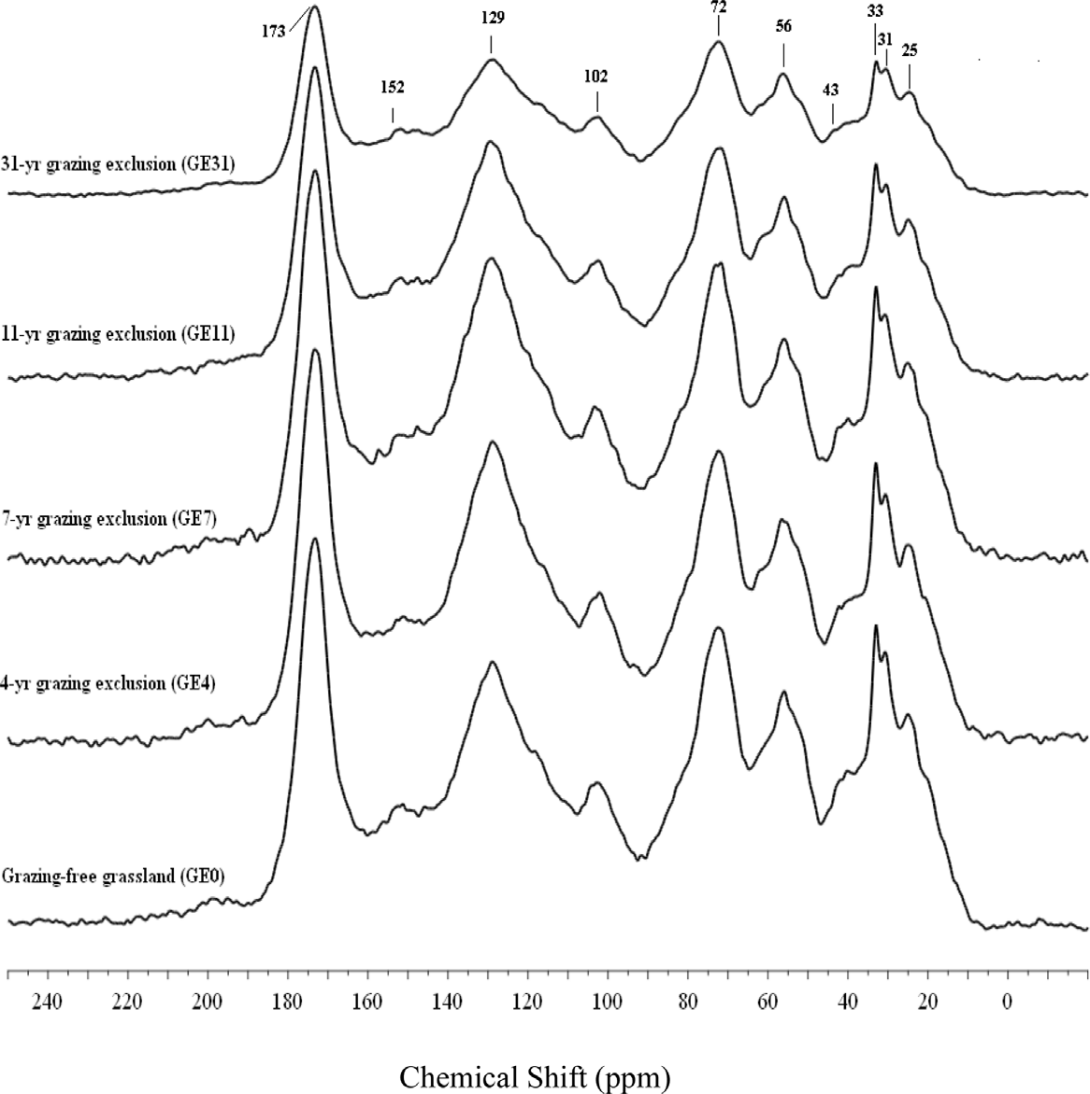
Solid-state^13^C CPMAS NMR spectra for humic acid (HA) under grazing-exclusion grassland chronosequence.

The structures of HA, as shown in Fig 2, were similar among the different plots. In detail, the contents of alkyl C and O-alkyl C first increased and then decreased with prolonged GE duration (Table 4). Aromatic C was lowest in GE0 (28.65%) and highest in GE4 (31.56%). The content of carbonyl C was significantly lower in GE31 than in other GE grasslands, but it was not significantly different among the plots of GE0, GE4, GE7, and GE11 (Table 4). The ratio of alkyl to O-alkyl decreased with GE, it was 0.03, 0.06, 0.04, and 0.04 in GE4, GE7, GE11, and GE31, respectively. The ratio of hydrophobic C to hydrophilic C increased with increasing GE duration, and reached relative equilibrium at decade of GE application.

**Table 4:** Relative distribution (%) of organic carbon in HA by ^13^C CPMAS NMR.

	Alkyl C (0–50ppm) (%)	O-alkyl C (50–110 ppm) (%)	Aromatic C (110–160 ppm) (%)	Carbonyl C (160–210 ppm) (%)	Aromaticity^‡^ (%)	Alkyl C/O-alkyl C	Hydrophobicity^§^
GE0^†^	19.33	34.45	28.65	17.57	0.35	0.56	0.92
GE4	17.84	33.45	31.56	17.15	0.38	0.53	0.98
GE7	17.39	34.45	31.44	16.72	0.38	0.50	0.95
GE11	17.64	34.04	30.69	17.64	0.37	0.52	0.94
GE31	18.66	35.57	29.82	15.95	0.35	0.52	0.94

^†^ GE0, free grazing; GE4, 4-year grazing exclusion; GE7, 7-year grazing exclusion; GE11, 11-year grazing exclusion; GE31, 31-year grazing exclusion.

^‡^ Aromaticity = Aromatic C/(Alkyl C+O-alkyl C+Aromatic C)×100%.

^§^Hydrophobicity = (Alkyl C + Aromatic C)/(O-alkyl C + Carbonyl C).

## Discussion

### Long-term grazing exclusion enhances soil C storage in semiarid grasslands

The content of SOC in the surface soil increased with GE, and the exponential equations well fitted the changes associated with the duration of GE. Our findings showed that SOC content in grasslands increased slowly in the first phase of GE, and faster after a decade of GE. The results were consistent with our previous study [30], suggesting that long-term GE can be conducive to enhancing SOC content. The change in SOC depended on the balance between SOM decomposition and new SOM input. The practice of GE promoted the restoration of grassland vegetation and directly resulted in increased SOM input from litter and roots [31,32]. In this study, the litter and aboveground biomass in these GE grasslands were significantly higher than those of grazing grasslands (Table 1). Additionally, the practice of GE decreased SOM decomposition by maintaining a better soil aggregate structure through exclusion of livestock stamping [33,34], and the higher height and density of the aboveground vegetation improved soil surface roughness, thereby reducing soil erosion by wind and water in these GE grasslands [35,36]. Furthermore, He et al. [37] reported that higher litter accumulation in the soil surface resulted in a lower soil temperature (2–3°C lower) in the long-term GE grasslands. Lower soil temperature may reduce the decomposition of SOM and benefit the accumulation of SOC to some extent [38].

### Long-term grazing exclusion improves soil humification and SOM stability

The composition of SOM varied among different plots, and long-term GE improved soil humification and SOM stability to some extent. Changes in SOM input characteristics (e.g., input, C/N ratio, and the content of protein and polysaccharides) and soil temperature and moisture may influence SOM breakdown and formation [34,39]. HAC increased logarithmically with the duration of GE (Fig 1B), that is, HAC increased initially and then, attained stability after a decade of GE. Dou [40] proposed that hypothermia decreased the formation of HA. Additionally, higher soil moisture could reduce SOM decomposition, and the reduced microbial activity could reduce the decomposition of HA. Moreover, excessive moisture will prevent further condensation of HA [41]. Therefore, lower soil temperature and higher moisture in the long-term GE grasslands [37] should be the main reasons for the alteration of HA. Sheng and Zhao [42] demonstrated that plant biomass and the content of HA were positively correlated in semi-arid habitat conditions because lower plant biomass and coverage in favor of higher O_2_ exchange between soil and atmosphere resulted in oxidative degradation of HA.

HUC increased exponentially with the duration of GE in Inner Mongolian grasslands. Yang et al. [43] found that an increase in the proportion of HA and HU in the presence of grass cover resulted in higher soil C sequestration potential. Moreover, Seddaiu et al. [11] demonstrated that the content of HUC can indicate the stability of SOM. Based on the findings that HUC and SOC have positive correlations in long-term GE grasslands, we assumed that the stability of SOM might be enhanced by long-term GE to some extent. In this study, the content of SOC and HUC increased exponentially with the duration of GE. The finding that HU and HUC did not arrive at the equilibrium after the 3-decade GE indicated that the recovery of recalcitrant fractions in addition to the total SOM pools requires a longer duration [44]. Thus, long-term GE not only increased SOM content but also made it more stable [4].Compared with FA, HA has higher molecular weight and the degree of polymerization, and the latter is associated with the humification rate. A higher HA/FA ratio indicates higher humification degree [45], and hence HA/FA is used as an index to determine soil humification degree and molecular complexity [46]. The ratios of HAC/extractable humus C have been used as an indicator for the degree of humification, where a higher ratio implies larger molecular weight, more complex molecular structure, and higher quality of HA [47]. The higher correlation of HAC/FAC and HAC/extractable humus C ratio reported here confirm that both measure represent the humification degree well. Furthermore, our findings that the ratios of HAC/FAC and HAC/extractable humus C increased exponentially with the duration of GE (Fig 1) imply higher degree of humification for the SOM.

### Grazing exclusion alters the composition of humic acid

HA is the most active component of humus, and its high cation exchange capacity enables soil fertilizer retention. It is also an organic binder that regulate the formation of soil structure [48]. A similar HA skeleton was observed in these GE grasslands (Fig 2), although there were some small alterations in the different components (Fig 2 and Table 4). Short-term GE decreased alkyl C and O-alkyl C and increased aromatic C. However, long-term GE increased the content of aliphatic C but decreased the content of aromatic C. Inconsistent changes in different HA components with the practice of GE led to the observed increases in aromaticity and the ratio of hydrophobic C/hydrophilic C, and the observed decrease in the ratio of alkyl C/O-alkyl C.

It was generally considered that alkyl C was derived from original plant biopolymers (such as cutin, suberin, and waxes) or from metabolic products of soil microorganisms, which comprise the most persistent fraction of SOM [49,50], whereas O-alkyl C (e.g., carbohydrates and polysaccharides) was easily decomposed; therefore, alkyl C/O-alkyl C is commonly used as an index of decomposability of SOM. The higher hydrophobicity of humic substances is indicates higher stability of SOM [51,52]. The ratio of aromaticity has been used to indicate the degree of aromaticity and aliphatic properties [53], with larger ratios indicating a more aromatic and less aliphatic humic substance. We therefore assumed that the soil structure in these long-term GE grasslands became more stable with stronger aliphatic properties and weaker aromaticity.

## Conclusion

Long-term GE significantly influences SOM composition. The contents of SOC, HUC, and the ratios of HAC/extractable humus C and HAC/FAC increase exponentially with the duration of GE, and HAC shows a significant logarithmic increase with prolonged GE. Based on the ratios of HAC/extractable humus C and HAC/FAC, we concluded that the humification degree increased in the 3-decade GE grasslands. Aromaticity, alkyl C/O-alkyl C ratio, and hydrophobicity decreased and HUC content increased in the long-term GE grasslands, which indicated the SOM was more stable. These findings provide new insights into the stability of increasing SOC storage in long-term GE grasslands in view of SOM composition and stability.

## References

[1] Tisdall JM, Oades JM (1982) Organic matter and water-stable aggregates in soils. Journal of Soil Science 33: 141–163.

[2] Breshears DD, Whicker JJ, Johansen MP, Pinder JE (2003) Wind and water erosion and transport in semi-arid shrubland, grassland and forest ecosystems: Quantifying dominance of horizontal wind-driven transport. Earth Surface Processes and Landforms 28: 1189–1209.

[3] Parton WJ, Schimel DS, Cole CV, Ojima DS (1987) Analysis of factors controlling soil organic matter levels in great-plains grasslands. Soil Science Society of America Journal 51: 1173–1179.

[4] Six J, Conant RT, Paul EA, Paustian K (2002) Stabilization mechanisms of soil organic matter: Implications for C-saturation of soils. Plant and Soil 241: 155–176.

[5] Stevenson FJ (1994) Humus chemistry: genesis, composition, reactions: John Wiley & Sons.

[6] Papini R, Valboa G, Favilli F, L’Abate G (2011) Influence of land use on organic carbon pool and chemical properties of Vertic Cambisols in central and southern Italy. Agriculture, ecosystems & environment 140: 68–79.

[7] Guimaraes DV, Gonzaga MIS, da Silva TO, da Silva TL, Dias ND, Matias MIS. (2013) Soil organic matter pools and carbon fractions in soil under different land uses. Soil & Tillage Research 126: 177–182.

[8] Piccolo A, Conte P, Spaccini R, Mbagwu J (2005) Influence of land use on the characteristics of humic substances in some tropical soils of Nigeria. European journal of soil science 56: 343–352.

[9] Watanabe A, Sarno, Rumbanraja J, Tsutsuki K, Kimura M (2001) Humus composition of soils under forest, coffee and arable cultivation in hilly areas of south Sumatra, Indonesia. European Journal of Soil Science 52: 599–606.

[10] Yang ZH, Singh BR, Sitaula BK (2004) Soil organic carbon fractions under different land uses in mardi watershed of Nepal. Communications in Soil Science and Plant Analysis 35: 615–629.

[11] Seddaiu G, Porcu G, Ledda L, Roggero PP, Agnelli A, Corti G. (2013) Soil organic matter content and composition as influenced by soil management in a semi-arid Mediterranean agro-silvo-pastoral system. Agriculture Ecosystems & Environment 167: 1–11.

[12] He N, Zhang Y, Dai J, Han X, Baoyin T, Yu G. (2012) Land-use impact on soil carbon and nitrogen sequestration in typical steppe ecosystems, Inner Mongolia. Journal of geographical sciences 22: 859–873.

[13] Zhou Z, Sun OJ, Huang J, Li L, Liu P, Han X. (2007) Soil carbon and nitrogen stores and storage potential as affected by land-use in an agro-pastoral ecotone of northern China. Biogeochemistry 82: 127–138.

[14] Wiesmeier M, Steffens M, Mueller C, Kölbl A, Reszkowska A, Peth S, et al (2012) Aggregate stability and physical protection of soil organic carbon in semi-arid steppe soils. European journal of soil science 63: 22–31.

[15] He NP, Yu Q, Wu L, Wang YS, Han XG (2008) Carbon and nitrogen store and storage potential as affected by land-use in a *Leymus chinensis* grassland of northern China. Soil Biology & Biochemistry 40: 2952–2959.

[16] He NP, Han XG, Yu GR, Chen QS (2011) Divergent changes in plant community composition under 3-decade grazing exclusion in continental Steppe. PlosOne 6(11): e26506 doi: 10.1371/journal. pone.0026506 22073169 10.1371/journal.pone.0026506 PMC3206806

[17] He NP, Wu L, Wang YS, Han XG (2009) Changes in carbon and nitrogen in soil particle-size fractions along a grassland restoration chronosequence in northern China. Geoderma 150: 302–308.

[18] Nelson D, Sommers L, Page A, Miller R, Keeney D (1982) Total carbon, organic carbon, and organic matter In: Page A.L., Miller R.H., and Keeney D.R., editors, Methods of soil analysis. ASA and SSSA, Madison, WI.

[19] Gallaher R, Weldon C, Boswell F (1976) A semiautomated procedure for total nitrogen in plant and soil samples. Soil Science Society of America Journal 40: 887–889.

[20] Kuo S (1996) Phosphorus In:DL et al (eds) Methods of soil analysis. Part 3. Chemical methods. Soil Science Society of America and American Society of Agronomy, Madison.

[21] Kumada K (1988) Chemistry of soil organic matter: Elsevier.

[22] Zhang JJ, Hu F, Li HX, Gao Q, Song XY, Ke X, et al (2011) Effects of earthworm activity on humus composition and humic acid characteristics of soil in a maize residue amended rice-wheat rotation agroecosystem. Applied Soil Ecology 51: 1–8.

[23] Lao J (1988) Handbook of Soil Agro-Chemistry Analysis. Beijing: Agriculture Publishing House.

[24] Piccolo A, Zaccheo P, Genevini P (1992) Chemical characterization of humic substances extracted from organic-waste-amended soils. Bioresource technology 40: 275–282.

[25] Dou S, Tan S, Xu X, Chen E (1991) Effect of pig manure application on the structural characteristics of humic acid in brown soil. Pedosphere 1: 345–354. 1647301

[26] Zhang JJ, Dou S, Song XY (2009) Effect of long-term combined nitrogen and phosphorus fertilizer application on 13C CPMAS NMR spectra of humin in a Typic Hapludoll of northeast China. European Journal of Soil Science 60: 966–973.

[27] Conte P, Piccolo A, van Lagen B, Buurman P, de Jager PA (1997) Quantitative differences in evaluating soil humic substances by liquid-and solid-state C-13-NMR spectroscopy. Geoderma 80: 339–352.

[28] Huan ZQ, Xu ZH, Chen CR, Boyd S (2008) Changes in soil carbon during the establishment of a hardwood plantation in subtropical Australia. Forest Ecology and Management 254: 46–55.

[29] Dai KH, Johnson CE, Driscoll CT (2001) Organic matter chemistry and dynamics in clear-cut and unmanaged hardwood forest ecosystems. Biogeochemistry 54: 51–83.

[30] Wu L, He N, Wang Y, Han X (2008) Storage and dynamics of carbon and nitrogen in soil after grazing exclusion in Leymus chinensis grasslands of northern China. Journal of Environmental Quality 37: 663–668. 18396553 10.2134/jeq2007.0196

[31] Gao YZ, Giese M, Lin S, Sattelmacher B, Zhao Y, Brueck H. (2008) Belowground net primary productivity and biomass allocation of a grassland in Inner Mongolia is affected by grazing intensity. Plant and Soil 307: 41–50.

[32] Gao Y, Giese M, Han X, Wang D, Zhou Z, Brueck H, et al (2009) Land use and drought interactively affect interspecific competition and species diversity at the local scale in a semiarid steppe ecosystem. Ecological research 24: 627–635.

[33] Steffens M, Kolbl A, Kogel-Knabner I (2009) Alteration of soil organic matter pools and aggregation in semi-arid steppe topsoils as driven by organic matter input. European Journal of Soil Science 60: 198–212.

[34] Wiesmeier M, Steffens M, Mueller CW, Kolbl A, Reszkowska A, Peth S, et al (2012) Aggregate stability and physical protection of soil organic carbon in semi-arid steppe soils. European Journal of Soil Science 63: 22–31.

[35] Hoffmann C, Funk R, Li Y, Sommer M (2008) Effect of grazing on wind driven carbon and nitrogen ratios in the grasslands of Inner Mongolia. Catena 75: 182–190.

[36] Hoffmann C, Funk R, Wieland R, Li Y, Sommer M (2008) Effects of grazing and topography on dust flux and deposition in the Xilingele grassland, Inner Mongolia. Journal of Arid Environments 72: 792–807.

[37] He N, Han X, Yu G, Chen Q (2011) Divergent changes in plant community composition under 3-decade grazing exclusion in continental steppe. PloS one 6: e26506 10.1371/journal.pone.0026506 22073169 PMC3206806

[38] He NP, Wang RM, Dai JZ, Gao Y, Wen XF, Yu GR. (2013) Changes in the temperature sensitivity of SOM decomposition with grassland succession: Implications for soil C sequestration. Ecology and Evolution 3: 10.1002/ece1003.1881 PMC3892367 24455135

[39] Doane TA, Devêvre OC, Horwáth WR (2003) Short-term soil carbon dynamics of humic fractions in low-input and organic cropping systems. Geoderma 114: 319–331.

[40] Dou S (2010) Soil organic matter: Beijing: Sciences Press.

[41] Peng F, Wu J (1965) Composition of humus in paddy soils. Acta Pedologica Sinica 13: 208–215.

[42] Sheng X, Zhao Y (1997) Impact of grassland biomass on soil organic matter. Chinese Journal of Soil Science 28: 244–245.

[43] Yang Z, Singh BR, Sitaula BK (2004) Fractions of organic carbon in soils under different crop rotations, cover crops and fertilization practices. Nutrient Cycling in Agroecosystems 70: 161–166.

[44] Burke IC, Lauenroth WK, Coffin DP (1995) Soil organic-matter recovery in semiarid grasslands: Implications for the conservation reserve program. Ecological Applications 5: 793–801.

[45] Yang Z, Singh B, Sitaula B (2004) Soil organic carbon fractions under different land uses in Mardi watershed of Nepal. Communications in soil science and plant analysis 35: 615–629.

[46] Doran JW (1980) Soil microbial and biochemical changes associated with reduced tillage. Soil Science Society of America Journal 44: 765–771.

[47] Dou S, Jiang Y (1988) Effect of application of organic materials on the properties of humic substances in organo-mineral complexes of soil—II. effect of organic materials on the humus composition and opticalcharacteristics of humic acids in organo-mineralcomplexes. Acta Pedologica Sinica 25: 252–261.

[48] Shang S (2012) Soil organic carbon fractions and their structures under different types of forest in subtropics: Zhenjiang A&F University.

[49] Dou S, Zhang JJ, Li K (2008) Effect of organic matter applications on ^13^C-NMR spectra of humic acids of soil. European Journal of Soil Science 59: 532–539.

[50] Ussiri DAN, Johnson CE (2003) Characterization of organic matter in a northern hardwood forest soil by C^-13^ NMR spectroscopy and chemical methods. Geoderma 111: 123–149.

[51] Piccolo A, Mbagwu JSC (1999) Role of hydrophobic components of soil organic matter in soil aggregate stability. Soil Science Society of America Journal 63: 1801–1810.

[52] Spaccini R, Mbagwu JSC, Conte P, Piccolo A (2006) Changes of humic substances characteristics from forested to cultivated soils in Ethiopia. Geoderma 132: 9–19.

[53] Zhang T, Li YF, Chang SX, Jiang PK, Zhou GM, Liu J, et al (2013) Converting paddy fields to Lei bamboo (Phyllostachys praecox) stands affected soil nutrient concentrations, labile organic carbon pools, and organic carbon chemical compositions. Plant and Soil 367: 249–261.

